# Identifying knowledge gaps for successful restorative aquaculture of *Ostrea edulis*: a bibliometric analysis

**DOI:** 10.12688/openreseurope.14074.3

**Published:** 2022-06-30

**Authors:** Camilla Bertolini, Roberto Pastres

**Affiliations:** 1 DAIS, Ca' Foscari University of Venice, Venice, 30170, Italy

**Keywords:** ecological requirements, European oysters, literature review, Ostrea edulis

## Abstract

**Background: **Active restoration is necessary to enhance the recovery of Ostrea *edulis* reefs, which contribute to many ecosystem services. Restoration can be integrated within aquaculture practices, bringing positive environmental changes while maximising space utilisation. The restoration project MAREA (MAtchmaking Restoration Ecology and Aquaculture) aims to bring back *O. edulis* in the North-West Adriatic addressing the feasibility of its cultivation. Both successful restoration and sustainable aquaculture require a thorough understanding of the ecological needs, as the requirements of both activities need to be harmonized. Therefore, one of the preliminary activities before embarking on the pilot was the completion of a thorough literature review to identify research directions and gaps required for ‘restorative aquaculture’, aiming to gather the most up to date O *. edulis *knowledge on a global and local scale.

**Methods: **Internet (Web of Science, Scopus, Google scholar) and physical resources (libraries) were searched for all available global and local knowledge on O *. edulis. Bibliometrix *was used to identify the main research topics using keywords, titles, and abstracts analyses. Studies were then manually screened and summarised to extract knowledge specific to restoration and aquaculture.

**Results: **While restoration studies are recent, evidence for the loss of this species and potential causes (and solutions) have been discussed since the end of the 19 ^th^ century. While diseases were a leading cause for reef loss, substratum limitation appears to be one of the leading limiting factors for both restoration and aquaculture of O *. edulis*, and was already mentioned in the early texts that were found.

**Conclusions: **The review highlighted that restoration success and aquaculture feasibility depend upon the crucial stage of settlement. The project ‘MAREA’ will therefore increase its focus on this stage, both in terms of timing, location, and materials for settlement plates placement.

## Introduction

Many benthic bivalve species are considered ecosystem engineers ( Jones *et al.*, 1994) and are involved in forming reefs, which are important habitats that support the biodiversity of marine ecosystems and contribute to multiple ecosystem services, including carbon accumulation ( Lee *et al.*, 2020; Lovelock & Duarte, 2019; van der Schatte Olivier *et al.*, 2020). These habitats, in particular oyster beds, are considered amongst the most degraded and imperilled, with 85% of natural reefs lost worldwide ( Beck *et al.*, 2011). Oysters are traditionally harvested for food. Aquaculture can be a solution to avoid overharvesting of natural populations, which can hinder the recovery of natural beds ( Smyth *et al.*, 2009).

There are over 200 species of oysters worldwide, but only 6 species belonging to four main genera ( *Crassostrea*, *Magallana*, *Ostrea* and *Saccostrea*) are currently being commercialised. Species of the genus *Ostrea* have the peculiarity of being ‘brooders’ or ‘partial spawners’ meaning that only males spawn and females brood eggs in their pallial cavity, whereas the other genus are complete spawners where both sexes spawn. This has some implications and reproduction success is dependent upon a threshold density ( Guy *et al.*, 2019). *Ostrea edulis,* the European flat oyster, was once widespread in the North Sea, on the Atlantic coast and in other European coastal waters, including the Mediterranean and Black Seas. However flat oysters became functionally extinct in wide areas, mainly due to overfishing, habitat loss, pollution, and disease: as a result, reefs are now one of the most threatened marine habitats in Europe. Culturing of this species is not new and has been practiced since the Roman times, particularly in Italy, which was the European leader of shellfish aquaculture until the 19th century. It is estimated that during the 1870s in the northern Adriatic Sea alone there was an annual production of 10 million oysters just from cultivation ‘parks’ ( Mattei & Pellizzato, 1997).

The Adriatic Sea is one of the shallower basins of the Mediterranean, with the northernmost part with an average depth of less than 100 m. It is also one of the areas boasting both the greatest invertebrate species diversity and the greatest risk from trawling and dredging and general exploitation of marine resources by fisheries ( Coll *et al.*, 2012). A historical study of the Adriatic Sea food-web and ecosystem functioning ( Lotze *et al.*, 2011) revealed that oyster reefs were pristine in the ‘pre-human’ period (before c.ca 100,000 BC) then became abundant during the ‘hunter-gatherer’ period (100,000 – 6,000 BC) until the classical period (500BC-600AD), when they were depleted, and eventually becoming rare in the ‘early global’ cultural period (1900–1950). In other areas, the loss of oyster beds was lamented in the early 19 ^th^ century, and suggestions were proposed for fostering the restocking, such as no harvesting during spawning season, resting ‘old’ beds for at least one year and helping the establishment of new beds with the use of cultch from a good bed ( Eyton, 1858). The human role in this loss is also highlighted in Mautner *et al.* (2018), which focused on the north-east Adriatic Sea ecosystem shifts and concluded that “ *while the mollusc community has changed continually over the past ~10,000 years and most of these changes have not been anthropogenically induced, the loss of vast Arca and Ostrea bottoms can be clearly linked to intensive and destructive fishing methods and other human-induced disturbances and regaining this special ecosystem, at least on a local scale, could be the goal of future restoration efforts”*.

Bivalve aquaculture has in recent years appeared as a solution for protein production with an emphasis on its sustainability ( Duarte *et al.*, 2009; Shumway *et al.*, 2003; Smaal *et al.*, 2019). More recently, terms such as ‘restorative aquaculture’ have made an appearance ( Carranza & Ermgassen, 2020). This approach may benefit all hierarchies of biodiversity, from the preservation of an imperilled species (e.g. *Ostrea lurida*, Ridlon *et al.*, 2021) to the recreation of habitat of value ( Theuerkauf *et al.*, 2021). Yet, being a new field, several of these consequences remain to be quantified, and the relative and absolute success of different strategies is yet to be assessed systematically. Successful restoration implies a deep understanding of the ecology of the target species, and of the historical baselines ( Basconi *et al.*, 2020; Thurstan *et al.*, 2020), as aspects such as location and timing of restoration projects can be essential to determine their success ( Cook *et al.*, 2021). MAREA aims at assessing the feasibility of restoring flat oyster reefs in the Northern Adriatic, by taking into account the ecological constraints and potential for improving the overall ecosystem functioning, as well as the economic benefits for shellfish farmers. The economic output and involvement of the aquaculture sector as a stakeholder in restoration should help securing financial resources to allow for large scale restorative actions ( Fitzsimons *et al.*, 2020).

Most research on restoration, so far, was done in northern America and northern Europe, with *Crassostrea virginica* dominating and *O. edulis* taking up only 1% of the overall research effort ( Toone *et al.*, 2021). The objective of this review is the identification of the knowledge gaps to be filled for achieving the goal(s) of MAREA, namely the set-up of an aquaculture practice that could be considered restorative. To this aim, the peer reviewed literature concerning *O. edulis* was screened using a two-step approach: 1) clusters of potentially relevant topics were identified using automated bibliographic tool which , based on a set of keywords ( Aria & Cuccurullo, 2017) and then 2) going into details of each cluster identified previously using both automated tools and manual searches to specifically focus on restoration and aquaculture. This helped to narrow the focus of the data gathering of the project MAREA, making optimal use of available knowledge to maximize the use of the funds available. Moreover, since the study by Toone *et al.* (2021) highlighted how only 2% of the knowledge is from traditional or indigenous sources, likely leading to an evidence base that is not reflective of the circumstances for most potential shellfish conservation projects, ultimately imperilling the success of their conservation work. This prompted the inclusion of an ‘historical and local knowledge’ section including both historical and modern sources was therefore added with searches in Italian that included ‘grey’ literature and alternative sources that could reflect site specific problematics that may be encountered in setting up a restorative aquaculture practice.

## Methods

### Global knowledge (quantitative)

R (version 4.0.5) was used ( R Development Core Team, 2021) for both automated and manual analyses of the selected literature.

The package *bibliometrix* was used for initial automated analyses. The procedures of ‘data collection’ highlighted in ( Aria & Cuccurullo, 2017) were followed. For data retrieval both Web of Science and Scopus were searched (all databases, last search 9/07/2021) using the keyword ‘ *Ostrea edulis*’. Titles and abstracts were then independently screened by the author (C.B.) to ensure that selected papers concerned this species. Only peer reviewed, English language articles were selected in this search. Any duplicate fields were removed. Author keywords were ranked based on their number of occurrences, after removal of words related to those directly ‘searched for’: ‘European oyster’, ‘flat oyster’, ‘ *Ostrea*’, ‘ *Ostrea edulis*’, ‘ostreidae’, ‘oyster’ (and plurals). Keywords that appeared in at least 10 papers were considered ‘popular’. Topic identification was aided by the use of ‘conceptualStructure’ firstly on the popular keywords (setting the minimum degree of occurrence to 10) with the dendrogram output used for interpretation of the word makeup of the different clusters to identify topics. Where possible, for each of the clusters, the algorithm was applied again, searching the titles and abstracts for common word (again setting the minimum degree of occurrence to 10) combination patterns. The ‘year’ field was extracted from the row names to calculate annual production and plot the number of articles per year.

### Global knowledge (qualitative)

Papers in each subcluster were then read and manually categorised, being assigned to a single category after reading. Some sub-categories were broken down into even smaller topics to get a clearer picture of the knowledge gaps by searching for specific words in keywords and titles and reading the articles. Furthermore, to specifically gather insights into articles on restoration and aquaculture the words ‘restoration’ and ‘aquaculture’ were searched for in keywords, titles and abstracts of the articles found to narrow the research question and understand if and how these two research areas differ in terms of research efforts. After reading the articles, these were then manually categorised into broad topics. Studies were summarised with particular attention to the identified environmental variables which were found to be favourable or unfavourable, the conclusions and recommendations for both restoration and aquaculture, to be taken into account in developing MAREA The geographical distribution of the studies was also considered.

### Historical and local knowledge (qualitative)

Internet resources (Web of Knowledge, Scopus and Google Scholar) were searched for ‘Ostrea *edulis*’ AND ‘Northern Adriatic’ OR ‘NW Adriatic’ OR ‘Adriatic lagoons’ OR ‘Venice’ OR ‘Venice lagoon’ and their Italian translations. Local library resources were also searched for any information available on oysters and molluscs, in particular their aquaculture, via the portal Bibliovea. Italian search terms were also included in this library search. Results that from this search yielded primarily reports and book chapters which were analysed qualitatively to extract information on the historical status of this species, its disappearance (timeline and causes) and any effort done on aquaculture and restoration, with a focus on environmental conditions tested and whether they were deemed as successes or failures.

## Results and discussion

### Global knowledge

**Figure 1.**
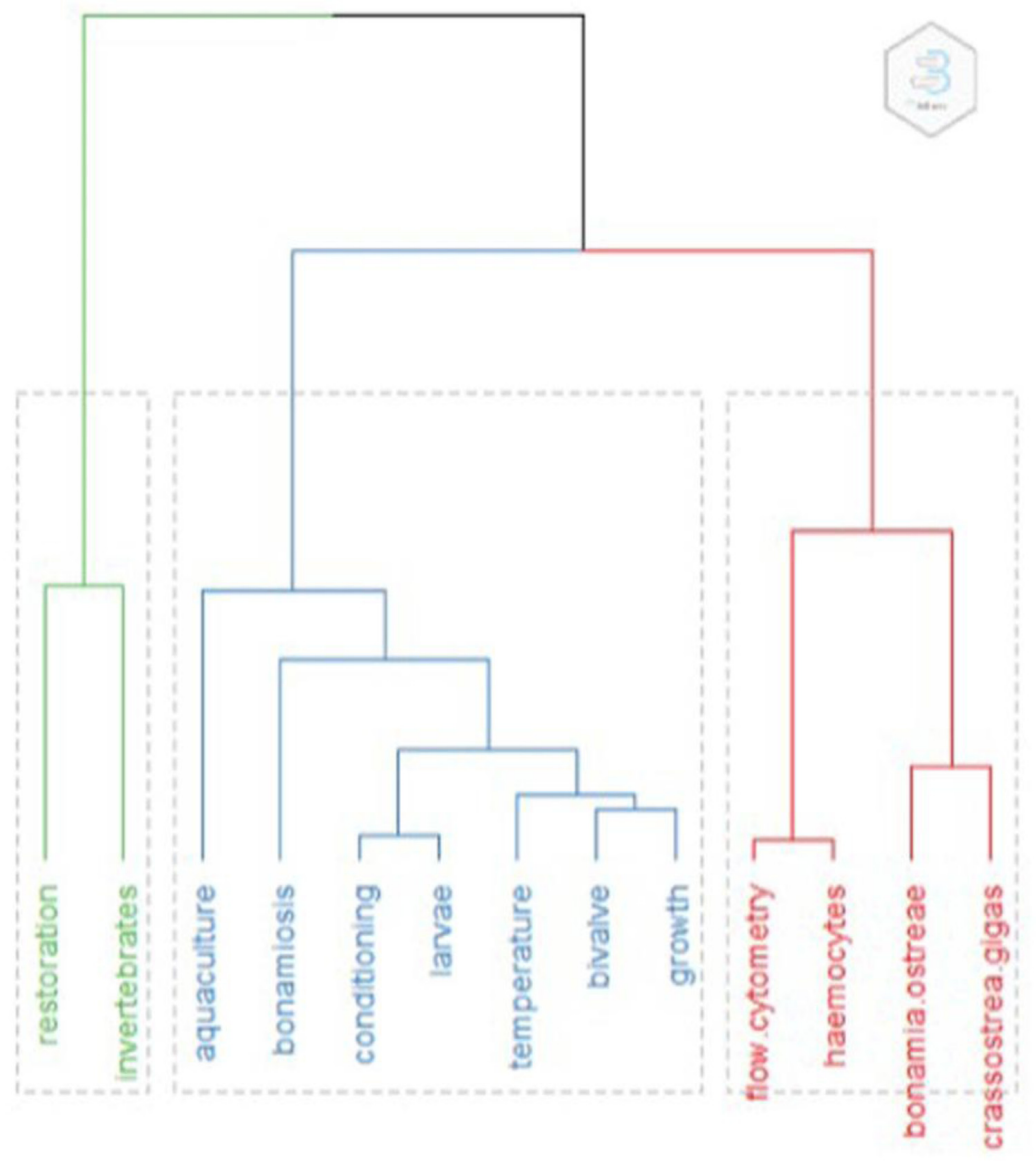
Clustering of keywords. Topic dendrogram generated by conceptual Structure, with the main words characterising each cluster.

The searches yielded 514 results of which 508 were classified as research articles and six were reviews (full reference list available as underlying data ( Bertolini & Pastres, 2021)). The first study was dated 1926, from which a few articles per year were produced until the 1960s, when the number started to increase to reach 35 articles in the year 2020. The number of publications related to restoration started to appear in the late 1990s and increased in the last five years, whereas research relating to aquaculture was emerging in the late 1970s, and while it followed the increasing trend, the overall output was generally lower ( Figure 1).

**Figure 2.**
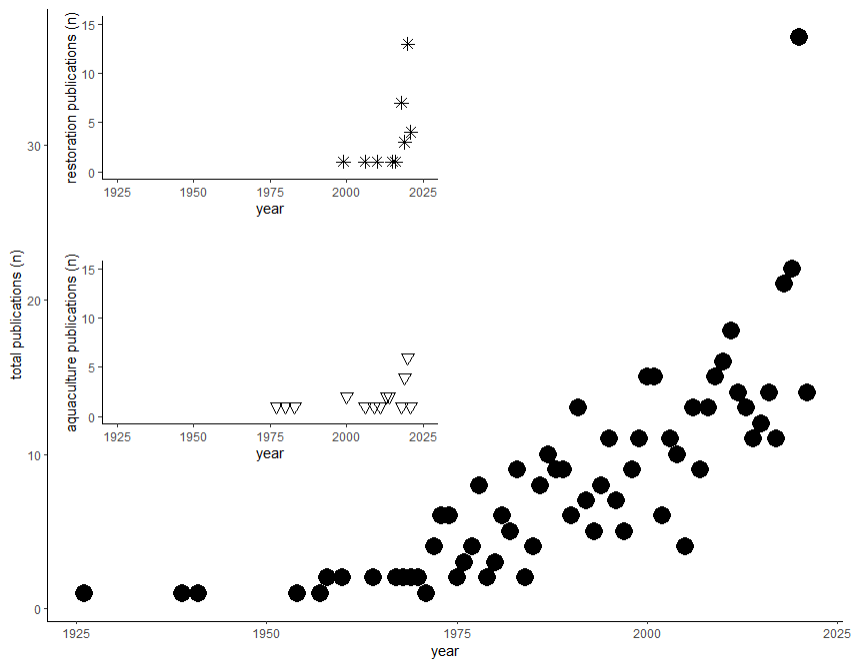
Number of publications per year. Main plot (full dots): total number of publications by year; top inset (stars): number of publications related to restoration by year; bottom inset (triangles): number of publications related to aquaculture by year.

The most prominent keywords found (N occurrences, %tot) were: *Bonamia ostreae* (52, 10%), *Crassostrea gigas* (28, 5.4 %), Restoration (21, 4%), Bivalve (16, 3%), Aquaculture (14, 2.7 %), growth (14, 2.7%), flow cytometry (12, 2.3%), temperature (12, 2.3 %), haemocytes (11, 2.1%), bonamiosis (10, 1.9%). A first interpretation of this result therefore is that infection appears as a highly studied aspect, with at least 13% of the studies concerned with infection from *Bonamia.* The clustering method used only 156 (30%) papers with common keyword associations, meaning that the remaining 70% of papers did not share important (appearing at least 10 times) terms in their keywords. Clusters are shown in the dendrogram in Figure 2. Words from subclusters are reported in Table 1.

**Table 1.** Subcluster characterization. Words in title, keywords and abstract characterizing each sub cluster.

Cluster	Subcluster	Words
1	N/A	Restoration, invertebrates
2	1	Biochemical, bonamia, conditioning, culture, development, effects, growth, implication, larval, rate, restoration, survival, temperature
	2	Crassostrea, gigas, magallana, native, pacific, species
	3	Adriatic, bay, larvae, sea
3	1	Bonamia, detection, haemocytes, parasite, resistance, vitro
	2	Haemocyte, haemolymph, infection, protozoan, stocks
	3	Bonamiosis, Crassostrea, gigas, response

Cluster one (green) had eight articles that dealt specifically with restoration. Sub-clustering was not possible due to the small number of papers Many aspects fell into this cluster: from the need of having high enough densities for successful reproduction ( Eagling *et al.*, 2018), and the seed collection stage ( Colsoul *et al.*, 2020; Rodriguez-Perez *et al.*, 2020; van den Brink *et al.*, 2020) to the suggestion that soft interventions such as protecting areas with a gravelly seabed may be a way to aid a natural come back in areas were a remaining natural population may be present even if in few numbers ( Kerckhof *et al.* (2018)). The need to consider infection in restoration procedures was also part of this cluster ( Sas *et al.*, 2020). Ecosystem effects of restoration such as the proper quantification of carbon deposition ( Lee *et al.*, 2020) and the missing questions that must be answered for the integration of policy and practice ( Zu Ermgassen *et al.*, 2020).

Cluster two (blue) had 93 articles. Title sub-clustering identified three subclusters: subcluster 1 papers related mostly to growth and larval development, stock, reproduction, settlement, the effects of temperature and food. Restoration and diseases also appeared as words in this cluster. Subcluster 2 papers were on comparisons or interactions between *C. gigas* and *O. edulis* regarding species distribution, biomarkers, biochemical and genetic assays, but also epibionts. A third subcluster was formed by a single paper on the seasonal distribution of larvae in the Adriatic Sea.

Cluster three (red) had 55 articles. Title sub-clustering identified three subclusters: subcluster 1 included papers on infection (Bonamiae), immunological activity and selection; subcluster 2 comprised papers on haemocyte parameters from different brood stocks in different geographic areas; and subcluster 3 papers were on the genetics of infections.

This analysis also gives some insights into the focus of the research concerning *Ostrea edulis*: the most numerous clusters contained papers mostly concerning the ecology of the species, while the second major cluster was formed by studies on infection and disease. Some interesting patterns arose on the main issues that may be considered by both restoration and aquaculture of this species. Infection, particular bonamiosis, may be an obstacle to both activities, and it is important to acknowledge that many studies are present including a comprehensive review ( Sas *et al.*, 2020). Primary subjects of *Bonamia* publications were: presence and transmission (22), resistance (19) methodologies of detection (12), responses and impacts (11), environmental variables (2). However, despite the high number of papers, both ( Sas *et al.*, 2020) and ( Holbrook *et al.*, 2021) underline a necessity to understand better the environmental variables that will be responsible for the infection and the mechanisms of resistance.

**Figure 3.**
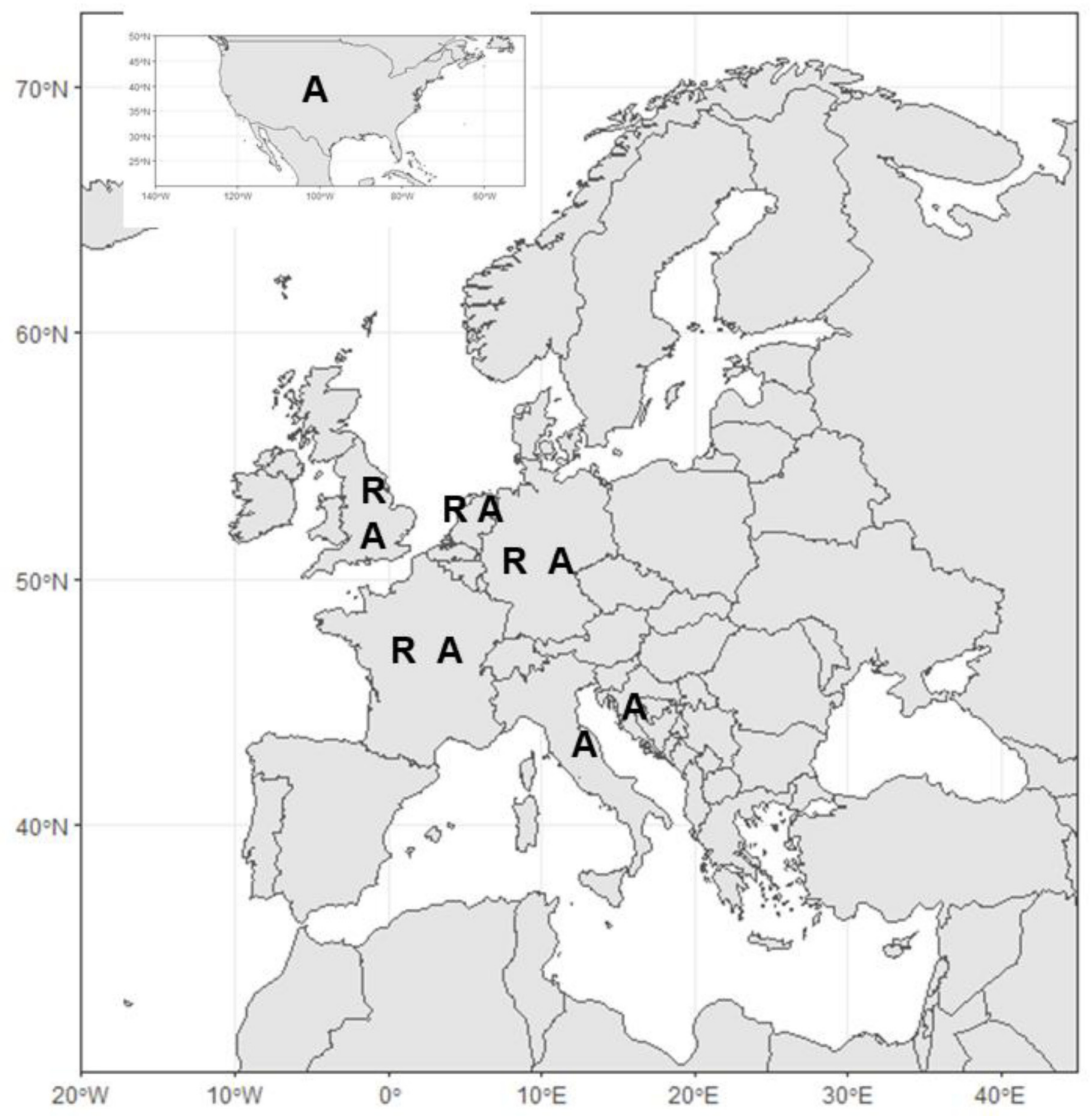
Annotated map representing countries where restoration (R) or aquaculture (A) studies are from.

In terms of this automated keyword association analysis, there appeared to be a few studies concerned solely with restoration. On the other hand, searching for restoration in keywords and titles manually yielded 35 articles, spanning from 1999 to 2021 ( Figure 2). Geographically, studies were primarily from Atlantic and North Sea regions (France, Germany, Netherlands, UK, Figure 3). Out of these, 12 (35%, 1999–2020) are concerned with ecological understanding, in particular related to site selection and conditions for growth, 10 (31%, 2018–2021) were related to settlement and seed production, four (12.5%, 2018–2020) regarded policy, three (9%, 2016–2019) were concerned with methodology for restoration, three (9%, 2018–2020) touched on the benefits of restoration, two (6%, 2020–2021) dealt with infection in restored oysters and only one (3%, 2010) dealt with genetic diversity.

Both the automatic clustering and manual search highlight how the ecological requirements that may be necessary to obtain a thriving and healthy population, thus towards ‘successful’ restorative aquaculture, are already widely studied. The restoration papers dealing with ecological understanding, for example, presented some agreements with regards to the value of the main environmental parameters necessary for success, as summed up in Table 2.

**Table 2.** Requirements for successful restoration.

Environmental parameter	Optimal value	References
Current speed	0.25–0.3 m/s	Kamermans *et al.*, 2018; Merk *et al.*, 2020; Pogoda *et al.*, 2020
Bottom shear stress	<0.3–0.4 N/m ^2^	Bennema *et al.*, 2020; Pogoda *et al.*, 2020
Sediment type	coarse grain size or presence of shell and stones for settlement	Allison *et al.*, 2020; Christianen *et al.*, 2018; Kamermans *et al.*, 2018; Pogoda *et al.*, 2020
	elevated cultch	Sawusdee *et al.*, 2015 (as seen for other species Marshall *et al.*, 1999; Wesson *et al.*, 1999)
Water temperature	7°C minimum for growth and gonad development	Maathuis *et al.*, 2020; Merk *et al.*, 2020
Oxygen	No agreement	
Chlorophyll	No agreement	

Searching for aquaculture in keywords and titles manually yielded 21 articles, spanning from 1977 to 2020 ( Figure 2). Geographically studies spanned both Atlantic and Mediterranean European regions (Croatia, France, Germany, Italy, Netherlands, UK, Figure 3), but there were also two studies concerning aquaculture of this species from the USA ( Burrell, 1983; Mann & Ryther, 1977). Of the two largest subgroups of studies, one dealt with the practicalities of seed production, including selective breeding (6, 28.5%), and the other with growth and biochemical composition of the marketable oyster product (5, 24%). There were also studies concerned with ecological conditions at the production site (2, 9.5%), with interactions with *C. gigas* (2, 9.5%), with infections (2, 9.5%) and with effects on the environment (2, 9.5%). Single studies were also done on history (1, 5%) and farm management from the human perspective (1, 5%).

Despite their different endpoints, in order to reach their goals both aquaculture and restoration must obtain healthy adults. This results in research perspectives that can be shared between the two ‘branches’. One common theme that emerged between the two was seed harvesting or production, an issue affecting aquaculture that relies mostly on wild seed collection but also affects restoration programmes that rely on active ‘seeding’ of often large quantities of oysters. Within this theme, two papers ( Colsoul *et al.*, 2021; van den Brink *et al.*, 2020) appeared in both searches. van den Brink *et al.* (2020) deals with the identification of the optimisation of collection, both in terms of collector types and methodology (e.g., timing), showing how ‘natural’ substrates (shell) would be optimal but raising questions related to the ‘economic viability’ of using this method for aquaculture purposes, which usually employs artificial collectors that simplify the process of detachment for the second phase of cultivation. Colsoul *et al.* (2021) provide a comprehensive review of seed production research in general, starting from the general biology of the species, identifying the stressors, and then looking at the history of production technologies, going into detail on seed production in polls, ponds and hatcheries. The review ends with a series of research gaps on the issue, such as the need to address the effects of climate change on reproductive patterns, something that is starting to be investigated in other bivalve species, e.g. mussels ( Oliveira *et al.*, 2021), but was already identified as a potential cause of *O. edulis* seed scarcity in 2015 ( Burioli *et al.*, 2015). This shows that an emerging field of ‘restorative aquaculture’ would need to address the issue of seed production, whether *in situ* or through hatcheries, ensuring enough seed can be collected or produced to ensure both economic viability and reefs creation/maintenance.

### Historical and local knowledge

With regards to oyster knowledge in the northern Adriatic Sea, multiple sources were found spanning the end of the 19 ^th^ and beginning of 20 ^th^ century. The most notable were two reports on the status of oyster culture in both the southern ( Molin, 1863) and northern ( Molin, 1864) parts of the Venice lagoon, a book on oyster and mussel culture ( Carazzi, 1893), and a thorough account of edible molluscs in the Venice lagoon with a whole chapter on oysters ( Ninni, 1904). More recent (end of the 20 ^th^ century) papers investigating settlement and culturing were also found ( Pellizzato & Da Ros, 1985; Pellizzato & Renzoni, 1986). Many of these examples already mention failed attempts, in particular related to obtaining successful reproduction (“ *a mistake in which many who attempted cultivating oyster fell into was to believe that to have successful spat would be enough to have some seawater, some adult oysters as mothers and some tiles to serve as collectors”* chapter XI ( Carazzi, 1893)). In the two reports from the 1860s, the ‘substrate’ limitation driving oyster reef self-sustainment was highlighted, and the dredging of hard material from the bottom of canals was pinpointed as one of the leading causes of the dramatic oyster loss observed in the lagoon. It was suggested that adding cleaned oyster shells could bring natural populations back, leading to the formation of ‘oyster parks’ ( Molin, 1863; Molin, 1864). The importance of location choice, collector specificity and timing, substratum type and environmental variables for the first stage of cultivation (seed harvesting) were already recognised as important ( Carazzi, 1893), together with the need for oyster culture to rely on trials and ‘naturalists’ advice ( Molin, 1863). The location of settlement, aside from having the right environmental conditions (for example a temperature warm enough to have sufficient spawning, even up to 28–30°C, but not higher to avoid mortality), would need to be close by to the location of culturing (at least for the first culturing phase, up to 3–4 cm) to avoid stressing the young oysters. The position of the collectors within the location was also already found to be an important issue: larvae were found to settle closer to the seabed, and for this reason, if collectors are on the seabed, it is important to ensure the sediment is neither too muddy to avoid sinking nor too sandy (indicative of too strong currents). The timing of collectors’ placement was also already identified in these early texts as an essential aspect that could be as important as the materials, as both too early and too late can have negative effects, either due to fouling by other organisms (if placed out too early) or due to missing the settlement period (when placing too late).

## Conclusions and perspectives

Articles concerning restoration are relatively new, mostly from the last five years, something already observed in other habitats (e.g. coastal wetlands ( Bertolini & da Mosto, 2021)), likely due to a surge in restoration activity in this period ( Duarte *et al.*, 2020) and it is possible that restoration will become the main discipline in ecological research ( Basconi *et al.*, 2020). When looking at the historical perspectives, however, it is evident that concerns and suggestions for restoration were already present. In the interest of maximising both time and resources, and avoid failures common within restoration frameworks ( Basconi *et al.,* 2020), the collated information on the ecological drivers, such as those presented in this review ( Table 2) regarding *Ostrea edulis*, should be used to draw restoration plans, coupled with local specificity and historical background of trials in the specific area where restorative aquaculture is to be set in place, which may require additional research in the grey literature, archives and other sources of local knowledge. 

Within MAREA, the ‘historical and local knowledge’ section of this paper led to the identification of a suitable area within the Venice lagoon to conduct the pilot and the design of the pilot itself had a heightened focus on the spawning and settlement stages, aiming to put to the test some of the solutions and suggestions found in the papers reviewed and developing models that would allow to scale up some of the results. In this study we noticed how local historical knowledge presented some similarities with recent, studies, pointing to the potential ubiquitousness of some of the issues related to substratum availability and how these, despite being known since the late 1800s, have not yet been resolved and may still be the main issue in the Venice Lagoon. However, local studies in other areas may present other issues as priority (e.g. temperature or current) and therefore it would be recommended to delve deeper into the historical reasons for disappearance and lack of natural re-establishment before embarking in restoration. Furthermore, the knowledge relating to seed production and harvesting learnt from academic and restoration trials, should be convened in appropriate manners, this means bridging linguistic gaps and jargon, to aquaculture practitioners, in order to make the two worlds, research and practice, coexist and limit the possibility of failed attempts.

Reproduction aspects, from gametogenesis and sex ratio, density of adults, larval transport and successful settlement, arose in this review as the most critical stage for both restoration and aquaculture What emerged in particular is the concern for the substrate limitation, which was already clear from the local historical perspectives and arises as one key issue also in the modern global restoration literature ( Bromley *et al.,* 2016; Chapman *et al.,* 2021; Rodriguez-Perez *et al.,* 2019; Smyth *et al.,* 2018). MAREA will therefore focus on the understanding of reproduction aspects, in particular its timing, larval dispersal and settlement on natural substrates (restoration) and collectors (aquaculture). Following the vast concern also for infection concerning both restorative and aquaculture practices, biosecurity protocols will be thoroughly followed throughout the whole MAREA project and transplanted oysters will be monitored closely also from a disease standpoint within the standard monitoring practices, which may enable to make links to environmental variables monitored in continuous.

Further, the account of optimal substrates in the historical literature seems, at least in part (‘not too muddy’), in accordance with current literature ( Table 2). This implies that the amount of knowledge present in what may be considered as ‘grey literature’ may be greater than imagined and already contains some essential information that could move restoration forward at a faster rate and with a greater chance of success. Of course, to access this it is important to access all repositories in multiple language and may require therefore international collaborations.

There are, however, potential issues surrounding restoration in general which remain unresolved although they do not specifically appear in the papers found in this review: specifically, the idea that protecting spaces may inhibit other uses. In the marine environment for example, the creation of new Marine Protected Areas (with a goal of having 30% of the sea protected by 2030) can lead to space use conflicts ( Knowlton, 2021). Having good legislation is necessary and correct maritime spatial planning designed to include restoration ( Lester *et al.*, 2020) can maximise space multi-functionality ( Schupp *et al.*, 2019), something possible in marine environments given they are three dimensional, providing conflict resolutions. In this context, the possibility to integrate aquaculture with restoration ( Giangrande *et al.*, 2021) can be a solution. This is what MAREA sets out to achieve, combining *O. edulis* restoration and seed production within existing mussel culture areas.

## Data availability

### Underlying data

Zenodo: Underlying data to: Identifying knowledge gaps for successful restorative aquaculture of Ostrea edulis: a bibliometric analysis. https://doi.org/10.5281/zenodo.5255971 ( Bertolini & Pastres, 2021).

Data are available under the terms of the Creative Commons Attribution 4.0 International license (CC-BY 4.0).
