# Supplementary material for: Identifying knowledge gaps for successful restorative aquaculture of Ostrea edulis: a bibliometric analysis
Source: Open Res Eur. 2022 Jun 30;1:103. Originally published 2021 Sep 6. [Version 3] doi: 10.12688/openreseurope.14074.3 (PMC10446074; doi:10.12688/openreseurope.14074.3)
Supplement: Peer review history

# Peer review: Reviewer response for version 3

**DOI:** 10.21956/openreseurope.16124.r29638

**Published:** 2022-07-13

**Authors:** Nadescha Zwerschke

**Affiliations:** 1 Greenland Climate Research Centre, Nuuk, Greenland

The authors have clearly addressed my concerns and should be congratulated on making their manuscript an easy and informative read.

Is the study design appropriate and does the work have academic merit?

Yes

Is the work clearly and accurately presented and does it cite the current literature?

Yes

If applicable, is the statistical analysis and its interpretation appropriate?

Yes

Are all the source data underlying the results available to ensure full reproducibility?

Yes

Are the conclusions drawn adequately supported by the results?

Partly

Are sufficient details of methods and analysis provided to allow replication by others?

Yes

Reviewer Expertise:

Benthic ecology with a focus on the role of oysters in temperate ecosystems and the impact of climate change in polar ecosystems.

I confirm that I have read this submission and believe that I have an appropriate level of expertise to confirm that it is of an acceptable scientific standard.

# Peer review: Reviewer response for version 3

**DOI:** 10.21956/openreseurope.16124.r29639

**Published:** 2022-07-01

**Authors:** Andrew Jeffs, Trevyn Toone

**Affiliations:** 1 Institute of Marine Science, School of Biological Sciences, University of Auckland, Auckland, New Zealand; 2 University of Auckland, Auckland, New Zealand

Great to see this tidied up further - thank you.

Is the study design appropriate and does the work have academic merit?

Partly

Is the work clearly and accurately presented and does it cite the current literature?

Partly

If applicable, is the statistical analysis and its interpretation appropriate?

Yes

Are all the source data underlying the results available to ensure full reproducibility?

Yes

Are the conclusions drawn adequately supported by the results?

No

Are sufficient details of methods and analysis provided to allow replication by others?

Yes

Reviewer Expertise:

NA

We confirm that we have read this submission and believe that we have an appropriate level of expertise to confirm that it is of an acceptable scientific standard.

# Peer review: Reviewer response for version 2

**DOI:** 10.21956/openreseurope.15579.r28342

**Published:** 2022-03-02

**Authors:** Andrew Jeffs, Trevyn Toone

**Affiliations:** 1 University of Auckland, Auckland, New Zealand; 2 Institute of Marine Science, School of Biological Sciences, University of Auckland, Auckland, New Zealand

Thank you for the opportunity to respond to the authors' revisions to the manuscript, arising from our earlier review. We appreciate the effort and detail the authors' have applied to improve the article, which has served to address the points we raised in our review. Thank you.

Is the study design appropriate and does the work have academic merit?

Partly

Is the work clearly and accurately presented and does it cite the current literature?

Partly

If applicable, is the statistical analysis and its interpretation appropriate?

Yes

Are all the source data underlying the results available to ensure full reproducibility?

Yes

Are the conclusions drawn adequately supported by the results?

No

Are sufficient details of methods and analysis provided to allow replication by others?

Yes

Reviewer Expertise:

NA

We confirm that we have read this submission and believe that we have an appropriate level of expertise to confirm that it is of an acceptable scientific standard.

# Peer review: Reviewer response for version 2

**DOI:** 10.21956/openreseurope.15579.r28343

**Published:** 2022-02-04

**Authors:** Nadescha Zwerschke

**Affiliations:** 1 Greenland Climate Research Centre, Nuuk, Greenland

In the updated version of Bertolini & Pastres, the authors provided a better introduction to the project they are planning to carry out. However, the purpose and usefulness of this paper remain unclear. As a review paper, it remains very short and lacking in detail regarding the outcomes of their findings, as an introduction and methods paper for the MAREA project it appears too generic in its findings. The results and discussions of the global section is, in fact, mostly a listing of their results from the word cluster analysis and I am missing a clear representation and discussion of the actual problem *O. edulis* restoration and aquaculture is facing. It appears questionable that the only main issue affecting aquaculture and restoration effort together is seed-production.

 Bertolini & Pastres include a section in the introduction highlighting that only 2% of knowledge regarding oyster conservation and restoration originates from indigenous sources Toone *et al*. (2021). Bertolini & Pastres go on to justify including a local knowledge section in their manuscript based on these findings by Toone *et al*. Whilst I do not debate that the Italian population is undoubtedly indigenous to Italy, I am wondering whether Toone *et al*. were defining indigenous in this context. I, generally don't see the need to justify the local knowledge section in such a way if its inclusion in the manuscript provides added value.

 As it stands, the local knowledge section is currently more enjoyable to read than the global knowledge section because it more clearly outlines some of the problems associated with restoration and aquaculture of *Ostrea edulis*. Yet, while problems identified in the local section confirm findings in other regions, these problems are generic and not necessarily specific to this region or have not been identified in other parts of the world, e.g. timings of collector placement, substratum type, dredging of habitats. What would be interesting for the MAREA project in the local knowledge section is a definition of a suite of environmental variables in which *O. edulis* thrive in the Venice lagoon and whether they are similar to those elsewhere in the world (e.g. Table 2). What are the times when *O. edulis* spawns there? Is that similar to times elsewhere in the world? Are there specific sites in the lagoon that local oyster farmers would go to, to collect seed? Why is that? Have there been significant decreases in the *O. edulis* populations and can they be linked to certain events - such as overfishing, increase in temperature, toxic algal blooms? Information like this would provide a much more robust justification for the inclusion of a local knowledge section.

 The conclusions and perspectives are still perplexing to the reader as the section draws out additional problems that have not been discussed extensively in the prior sections and comes to the conclusion that international collaborations might be required to evaluate further grey literature. It also touches on the problematic of establishments of MPAs and marine spatial planning that feel out of place in this section. Similarly, the section where biosecurity protocols of the MAREA project are being discussed could be removed from the manuscript.

 A general expectation for a conclusion and perspective section of a review would be a summary of your main findings (e.g. the main issues with *O. edulis* restoration and aquaculture) and how to take them further as part of your project. From my perspective, this has not yet been achieved here. The manuscript would also benefit from a careful spell and grammar check.

Is the study design appropriate and does the work have academic merit?

Yes

Is the work clearly and accurately presented and does it cite the current literature?

Yes

If applicable, is the statistical analysis and its interpretation appropriate?

Yes

Are all the source data underlying the results available to ensure full reproducibility?

Yes

Are the conclusions drawn adequately supported by the results?

Partly

Are sufficient details of methods and analysis provided to allow replication by others?

Yes

Reviewer Expertise:

Benthic ecology with a focus on the role of oysters in temperate ecosystems and the impact of climate change in polar ecosystems.

I confirm that I have read this submission and believe that I have an appropriate level of expertise to confirm that it is of an acceptable scientific standard, however I have significant reservations, as outlined above.

# Response

**Published:** 2022-06-23

**Authors:** Camilla bertolini

**Affiliations:** Ca' Foscari University of Venice, Italy

We are thankful to the reviewer for their comments.

 We reply to all of the comments in one section in order from the introduction to the conclusion.

 The aim of this research, as expressly mentioned in the abstract is that of  'identify research directions and gaps required for ‘restorative aquaculture’' We now added more information on the goals at the end of the introduction.

 Toone *et al*. mentions 'traditional or indigenous' sources, we added 'traditional' but did 

 not remove this section as believe it is important to consider "alternative" sources of knowledge. The sentence was expanded for clarity, and while we are aware that we do not have 'indigenous' tribes the knowledge of those not directly involved in research, or hard to find (e.g. only library holdings) may be ignored if not actively searched for:

 'This prompted the inclusion of an ‘historical and local knowledge’ section including both historical  and modern sources was therefore added with searches in Italian that included ‘grey’ literature and alternative sources  that could reflect site specific problematics that may be encountered in setting up  a restorative aquaculture practice.'

 In terms of the results, we understand that they may feel different from those of a classical review, but we felt the need to present the results from the clustering analysis in a quantitative format and the paper is presented as a research paper instead of a review *per see*. In this new version we added some more details in the results and discussion, particularly to 'cluster 1' concerned with restoration, where details of all papers clustered within this were added.

 The keywords were also added to the table.

 The 'local' knowledge was renamed 'historical and local' and the conclusions highlight how was this section that allowed us to choose the site (the Venice lagoon) to set up the pilot within the Adriatic Sea, hoping to prompt more consideration for historical literature and the necessity to consult it where possible prior to restoration efforts.

 The conclusion section was restructured and now includes more details on the issues found and how the project will aim to answer them:

 ‘Reproduction aspects, from gametogenesis and sex ratio, density of adults, larval transport and successful settlement, arise as the most critical stage for both restoration and aquaculture. What emerges in particular is the concern for the substrate limitation, which was already clear from the local historical perspectives and arises also as one key issue also in the modern global restoration literature ( Bromley et al., 2016; Chapman et al., 2021; Rodriguez-Perez et al., 2019; Smyth et al., 2018). MAREA will therefore focus on the understanding of reproduction aspects, in particular its timing, larval dispersal and settlement on natural substrates (restoration) and collectors (aquaculture).  ' 

 Regarding the aspects of spatial planning that arise in the conclusions and perspectives, we now clearly state that these are potential issues for restoration in general, considered in other cases, that do not appear in the findings of the review, but may be considered in the context of 'future perspectives', and was moved towards the bottom of the paragraph.

# Peer review: Reviewer response for version 1

**DOI:** 10.21956/openreseurope.15169.r27967

**Published:** 2021-11-19

**Authors:** Nadescha Zwerschke

**Affiliations:** 1 Greenland Climate Research Centre, Nuuk, Greenland

Bertolini & Pastres' manuscript is a short literature review aiming to address knowledge gaps associated with the potential restoration of the European Oyster, *Ostrea edulis*, in the North-West Adriatic. This literature review is being carried out to better direct the approach of a new research project - MARESA (Matchmaking Restoration Ecology and Aquaculture) to the purpose of oyster restoration. The methodology of the review was thorough and the analysis was carried out well. Discussion of results was sparse and I do not necessarily feel there was a clear identification of research gaps throughout the discussion or conclusion, which was a lost opportunity.

**Introduction:**

 The introduction is well written, it might be useful to the reader to introduce the MARESA project to help understand the link between restoration and aquaculture you are trying to make throughout the manuscript.

**Methods:**

 The methods are generally clear. It would be interesting to know why the authors have opted not to use Google Scholar for their search on global knowledge.

**Results and Discussion:**

- **Global knowledge:**
  There is little to no discussion of the results that have been gained. This part of the manuscript contains a short paragraph where some common findings regarding *O. edulis* aquaculture amongst the literature are starting to become more defined. However, the authors neglect to take these findings further and clearly specify research gaps based on these literature findings.
- **Local knowledge:**
  Although interesting, I am unsure whether it is necessary to recapitulate historic aspects of aquaculture in the northern Adriatic. The need for appropriate habitat and settlement substratum is also not a problem specific to the Adriatic or *Ostrea edulis,* but one that most regions across the globe are facing when attempting to restore oyster beds. No clear research gaps or directions specific to this region are being identified in this part of the manuscript.
- **Conclusions:**
  The conclusions suffer from the fact that the results and discussion have not really revealed any clear research questions to be addressed in the future. Thus, it is surprising to me that the authors decided to focus on the settlement aspect of the oysters - an area which is, according to the authors (93 papers in the cluster) comparatively well researched.
  I do not doubt that this is an important part of the oyster growth process, yet the manuscript would improve if the need for research in these areas would be made clearer throughout.

**Table 1:** Consider including number of papers per keyword to provide a clear representation of research effort in the different categories.

**Figure 1:** I am unsure about the need for this figure, it might be worth thinking about including a figure representing the comparative research effort (e.g. number of papers) for different subcategories (e.g. aquaculture, disease, competition etc.) instead.

Is the study design appropriate and does the work have academic merit?

Yes

Is the work clearly and accurately presented and does it cite the current literature?

Yes

If applicable, is the statistical analysis and its interpretation appropriate?

Yes

Are all the source data underlying the results available to ensure full reproducibility?

Yes

Are the conclusions drawn adequately supported by the results?

Partly

Are sufficient details of methods and analysis provided to allow replication by others?

Yes

Reviewer Expertise:

Benthic ecology with a focus on the role of oysters in temperate ecosystems and the impact of climate change in polar ecosystems.

I confirm that I have read this submission and believe that I have an appropriate level of expertise to confirm that it is of an acceptable scientific standard, however I have significant reservations, as outlined above.

# Response

**Published:** 2022-01-07

**Authors:** Camilla bertolini

**Affiliations:** Ca' Foscari University of Venice, Italy

Reviewer comments in italics throughout.

*Bertolini & Pastres' manuscript is a short literature review aiming to address knowledge gaps associated with the potential restoration of the European Oyster, Ostrea edulis, in the North-West Adriatic. This literature review is being carried out to better direct the approach of a new research project - MARESA (Matchmaking Restoration Ecology and Aquaculture) to the purpose of oyster restoration. The methodology of the review was thorough and the analysis was carried out well. Discussion of results was sparse and I do not necessarily feel there was a clear identification of research gaps throughout the discussion or conclusion, which was a lost opportunity.*

*Introduction:*

*The introduction is well written, it might be useful to the reader to introduce the MARESA project to help understand the link between restoration and aquaculture you are trying to make throughout the manuscript.*

**Author Response: **We agree with the reviewer that the project could have been better introduced, we have now included further details in the introduction hoping the needs for the link between aquaculture and restoration are made clearer:

 ‘MAREA aims at assessing the feasibility of restoring flat oyster reefs in the Northern Adriatic, by taking into account  the ecological constraints and potential for improving the overall ecosystem functioning , as well as the  economic benefits for shellfish farmers. The economic output and involvement of the aquaculture sector as a stakeholder in restoration should help securing financial resources to allow for large scale restorative actions (Fitzsimons *et al*., 2020)’

*Methods:*

*The methods are generally clear. It would be interesting to know why the authors have opted not to use Google Scholar for their search on global knowledge.*

**Author Response: **This is because bibliometrix, used for the quantitative analysis, reads in data directly from the databases, and Google Scholar outputs would not have been compatible. For this part only peer reviewed English articles were chosen, therefore by using both databases we believe we would have found all relevant research.

*Results and Discussion: Global knowledge:*

*There is little to no discussion of the results that have been gained. This part of the manuscript contains a short paragraph where some common findings regarding O. edulis aquaculture amongst the literature are starting to become more defined. However, the authors neglect to take these findings further and clearly specify research gaps based on these literature findings.*

*Local knowledge:*

*Although interesting, I am unsure whether it is necessary to recapitulate historic aspects of aquaculture in the northern Adriatic. The need for appropriate habitat and settlement substratum is also not a problem specific to the Adriatic or Ostrea edulis, but one that most regions across the globe are facing when attempting to restore oyster beds. No clear research gaps or directions specific to this region are being identified in this part of the manuscript.*

*Conclusions:*

*The conclusions suffer from the fact that the results and discussion have not really revealed any clear research questions to be addressed in the future. Thus, it is surprising to me that the authors decided to focus on the settlement aspect of the oysters - an area which is, according to the authors (93 papers in the cluster) comparatively well researched. I do not doubt that this is an important part of the oyster growth process, yet the manuscript would improve if the need for research in these areas would be made clearer throughout.*

**Author Response:** We have addressed the points related to global knowledge, local knowledge and conclusions together as they all shared common themes, we added text throughout hoping to have clarified and emphasized some of the issues rightly highlighted by the reviewer. In specific, some details were added into the introduction as to the reasons why indigenous/local knowledge and historical context is important:

 ‘Moreover, since the study by Toone *et al.* (2021) highlighted how only 2% of the knowledge is from indigenous sources, likely leading to an evidence base that is not reflective of the circumstances for most potential shellfish conservation projects, .ultimately imperilling the success of their conservation work. A local knowledge section including both historical and modern sources was therefore added with searches in Italian. ’

 We also added some details in the results and discussion section in order to highlight some of the specificity of this review that relates to restorative aquaculture and gaps that need to be addressed for this emerging field:

 ‘Despite their different endpoints, in order to reach their goals both aquaculture and restoration must obtain healthy adults. This results in research perspectives that can be shared between the two “branches”’ \ ‘This shows that an emerging field of “restorative aquaculture” would need to address the issue of seed production, whether in situ or through hatcheries, ensuring enough seed can be collected or produced to ensure both economic viability and reefs creation/maintenance.’

 A whole part of analysis was added related to the studies concerning Bonamiosis, which were also sub-categorised as this topic is of high interest for both restoration and for aquaculture. The conclusion section was supplemented adding:

- ‘What emerges in particular is the concern for the substrate limitation which was already clear from the local historical perspectives and arises also as one key issue in the modern global restoration literature’
- ‘Further, the account of optimal substrates in the historical literature seems, at least in part (not too muddy), in accordance with current literature (table 2). This implies that the amount of knowledge present in what may be considered as ”grey literature” may be greater than imagined and already contain some essential information that could move restoration forward at a faster rate and with a greater chance of success. Of course, to access this it is important to access all repositories in multiple language and may require therefore international collaborations.’
- ‘Within MAREA, this led to the identification of a suitable area within the Venice lagoon to conduct the pilot and the design of the pilot itself had a heightened focus on the spawning and settlement stages, aiming to put to the test some of the solutions and suggestions found in the papers reviewed and developing models that would allow to scale up some of the results. Biosecurity protocols will be thoroughly followed throughout the whole MAREA project, to avoid any issues with diseases, thus despite the findings in this review, which identified some knowledge gaps in the understanding of their spreading, it may not be possible to address these within the study. The transplanted oysters will be monitored closely also from a disease standpoint within the standard monitoring practices, which will enable to make links to environmental variables monitored in continuous.’
- ‘In this study we noticed how local historical knowledge presented some similarities with recent, more globalised, studies, pointing to the potential ubiquitousness of some of the issues related to substratum availability and how these, despite being known since the late 1800s, have not yet been resolved and may still be the main issue in the Venice Lagoon, the area where MAREA is taking place. However, local studies in other areas may present other issues as priority (e.g. temperature or current) and therefore it would be recommended to delve deeper into the historical reasons for disappearance and lack of natural re-establishment before embarking in restoration.  Furthermore, the knowledge relating to seed production and harvesting learnt from academic and restoration trials, should be convened in appropriate manners, this means bridging linguistic gaps and jargon,  to aquaculture practitioners, in order to make the two worlds coexist and limit the possibility of failed attempts.’  

*Table 1: Consider including number of papers per keyword to provide a clear representation of research effort in the different categories.*

**Author Response: **The papers for the main number of keywords are presented already in text together with a percentage of the total output:

  ‘The most relevant keywords (N occurrences, %tot) were: Bonamia ostreae (52, 10%), Crassostrea gigas (28, 5.4 %), Restoration (21, 4%), Bivalve (16, 3%), Aquaculture (14, 2.7 %), growth (14, 2.7%), flow cytometry (12, 2.3%), temperature (12, 2.3 %), haemocytes (11, 2.1%), bonamiosis (10, 1.9%).’

 ​​​​​​​

*Figure 1: I am unsure about the need for this figure, it might be worth thinking about including a figure representing the comparative research effort (e.g. number of papers) for different subcategories (e.g. aquaculture, disease, competition etc.) instead.*  

**Author Response: **Figures were moved around so that figure 1 is now figure 2 as this figure is linked with the second part which investigates restoration and aquaculture outputs separately.

# Peer review: Reviewer response for version 1

**DOI:** 10.21956/openreseurope.15169.r27556

**Published:** 2021-10-01

**Authors:** Andrew Jeffs, Trevyn Toone

**Affiliations:** 1 Institute of Marine Science, School of Biological Sciences, University of Auckland, Auckland, New Zealand; 2 University of Auckland, Auckland, New Zealand

In their manuscript “Identifying knowledge gaps for successful restorative aquaculture of *Ostrea edulis*: a bibliometric analysis” Camila Bertolini and Roberto Pastres seek to identify research gaps in the existing global and local literature surrounding *Ostrea edulis*, ultimately informing restorative aquaculture projects like MAREA. The concept of the review is compelling and the methods are generally sound, however, the review would greatly benefit from additional information throughout the manuscript. In particular, the introduction would benefit by providing additional background on the study species, MAREA, existing literature reviews, and the specific goals of the project. The results also need clarification around the clustering and subcategorization process. Additionally, the methodology around clustering and keyword analysis needs more information to demonstrate that it is an appropriate study design for this project. Finally, the current conclusions of the review are quite vague. Explicit and detailed recommendations would be very useful to future efforts in this field. Overall, the review has good potential, but the current conclusions and methodology are not supported well enough for the work to have sufficient rigour and ultimately impact for readers and end users.

**Title and keywords:**

 The title is informative and describes the focus of the review well. Consider replacing the *Ostrea edulis *keyword as it is already present in the title and will be picked up by search engines.

**Abstract:**

 The connection between the results and the conclusion is unclear in this abstract. I recommend clarifying the conclusions and more clearly establish the connection to the results.

**Introduction:**

 Further clarification is needed on exactly what “research gaps required for restorative aquaculture” this literature review is targeting. The previous sentence suggests a focus on either ecological gaps (how and where restorative aquaculture can be conducted) or historical baseline gaps (extent of historical populations), but the sentence prior to that suggests a focus on gaps around output quantification (“the relative and absolute success of different strategies”). Is the intention to search for information on all of these gaps or only a narrower scope?

 Brief background information on *Ostrea edulis *would be very helpful in either in the introduction or the beginning of the methods section. This oyster has some unusual aspects to its biology which are particularly pertinent to restoration initiatives. Likewise, some detail of the nature of the biogenic habitat formed by this species would be valuable, and the geographic range of the species.

 Additional information on the methods used here (particularly keyword tracking) would be useful in the introduction. Why is this method being used and what information can be garnered from it?

 Additional background on existing literature reviews of *Ostrea edulis* research would provide useful information on gaps already known to exist in the literature. *The benefits of bivalve reef restoration: A global synthesis of underrepresented species *(zu Ermgassen et al., 2020 ^1^) documents the ecosystem services provided by *Ostrea edulis* after a systematic review of the literature while *Conserving shellfish reefs – A systematic review reveals the need to broaden research efforts *(Toone et al., 2020 ^2^) reports on common restoration interventions undertaken for shellfish reefs including *Ostrea edulis*.

 MAREA is mentioned in the abstract and conclusion, but never actually introduced anywhere in the manuscript, which seems a lost opportunity. Consider adding information on this project to the introduction.

**Methods:**

 Clarification is needed in the fifth paragraph on whether ‘restoration’ and ‘aquaculture’ were ran through Web of Science and Scopus as new search terms or whether the already identified papers were then further narrowed down by these search terms.

 Clarification on how the “broad topics” articles used for article sorting were developed would be useful. Were they pre-defined or developed after reading?

 Why is the keyword clustering method being used? What information does it present that helps address the goals of this review?

 Were repeats present between the global knowledge and local knowledge searches? The local knowledge database search appears to just be a more narrow repeat of the same search conducted earlier (any papers with the keywords ‘ *Ostrea edulis*’ and one of the location specific terms should have already shown up under just ‘ *Ostrea edulis*’).

 Was the ‘local knowledge’ database search also conducted in Italian to initiate new results or only the library search?

 How were the papers that resulted from the ‘local knowledge’ search analysed? Identically to the global knowledge results or using different methods? This information should be added to the ‘Local knowledge’ subsection.

**Results and discussion:**

 As a whole this section is very light on discussion and could benefit from a more in-depth insight into some of the implications of these findings. Separate results and discussion sections may prove useful.

 The geographic results of this review are interesting, but difficult to ascertain in their current form. What are the numbers behind “Geographically, studies were primarily from Atlantic and North Sea regions” or “studies spanned both Atlantic and Mediterranean European regions”? A map of included studies as a figure may be a very useful addition in this regard.

 The connection between Figure 2 and Table 1 is unclear. How do the subclusters identified in Table 1 correlate with the clades in Figure 2? For example, there’s only two clear clades in cluster three (red) but three subclusters. How do the terms used to identify the leaves in the dendogram correlate with the words characterizing each subcluster? More information on these connections would be very informative.

 Discussion on how the identified clustering informs restorative aquaculture decisions is needed. Where are the current gaps in the research? What does the clustering tell us about how to move forward with restorative aquaculture projects?

 Why was the clustering method only used for 156 out of the 514 papers? Did the remaining papers have no common terms? Clarification would be useful.

 There are 32 identified restoration articles, but the more detailed breakdown in the seventh paragraph reports 35 articles. Is this because a single article could be categorized into multiple themes? Did only three articles fit into multiple categories? Clarification in the methods section would be helpful.

 The information presented in the seventh paragraph identifying ecological parameters for successful populations is very useful. Consider making this into a table to emphasize these findings and allow for easier interpretation.

 Was it possible for the aquaculture papers to be subcategorized into multiple groups in the eighth paragraph and none were or was each paper intentionally only assigned a single subcategory?

 The local knowledge section is very informative. Consider making additional comparisons between this historical local knowledge and the results of the current review as there seem to be interesting similarities and differences.

**Conclusions:**

 This conclusion section is very brief and would benefit greatly from additional details.

 The conclusion states “…the collated information on the ecological drivers, such as those presented in the review regarding *Ostrea edulis *should be used…” but does not address how this information should be used.

 These results “led to the identification of a suitable area within the Venice lagoon to conduct the pilot” for MAREA, but the conclusion does not state which results led to this ability or how they were used. Were suitable areas compared with the ecological information? Was the local knowledge used to identify historical areas that could be used? How could another project use this information in a similar way?

 Finally, the conclusion recommends that “this knowledge should be convened in appropriate manners to aquaculture practitioners, bridging linguistic gaps, in order to make the two worlds coexist and limit the possibility of failed attempts.” How would the authors suggest this is done? What are the “appropriate manners” and what exactly is the knowledge the authors are suggesting is shared? This review provides a lot of interesting results, but these recommendations need to be streamlined to clarify how the authors think this information will be more useful.

**Figures and tables:**

 Consider adjusting the y-axes of the two inset graphs in Figure 1 to be identical (0-15). In the figure’s current form it appears the aquaculture and restoration subtopics contain similar numbers of articles, when aquaculture actually includes fewer articles than restoration but has a smaller scale on the y-axis.

 Does the “Height” y-axis of Figure 2 correlate to any real values? I would suggest simply removing this axis.

**Typos:**

 Abstract under the results subsection: “While diseases *was* a leading cause for reef loss…” should be “While diseases *were* a leading cause for reef loss”

 First sentence of introduction: “…which are important habitats *supporting* the biodiversity of marine ecosystems and *contribute* to multiple ecosystem services…” should either be “…which are important habitats *that support* the biodiversity of marine ecosystems and contribute to multiple ecosystem services…” or “…which are important habitats supporting the biodiversity of marine ecosystems and *contributing* to multiple ecosystem services…”

 First sentence of the third paragraph of the introduction: “Bivalve aquaculture has in recent years returned in the first line with an emphasis on sustainability.” What is the meaning of “returned in the first line”?

 The placement of the “(Figure 1)” citation in the first paragraph of the results section is unusual and breaks up the sentence. Consider moving it to the end of the sentence.

**References:**

 Burrell (1983) is entirely capitalized.

 Check capitalization consistency throughout references as some use title case and others use sentence case.

 Jones (1994) has extra word “organisms”.

Is the study design appropriate and does the work have academic merit?

Partly

Is the work clearly and accurately presented and does it cite the current literature?

Partly

If applicable, is the statistical analysis and its interpretation appropriate?

Yes

Are all the source data underlying the results available to ensure full reproducibility?

Yes

Are the conclusions drawn adequately supported by the results?

No

Are sufficient details of methods and analysis provided to allow replication by others?

Yes

Reviewer Expertise:

NA

We confirm that we have read this submission and believe that we have an appropriate level of expertise to state that we do not consider it to be of an acceptable scientific standard, for reasons outlined above.

## References

[1] : The benefits of bivalve reef restoration: A global synthesis of underrepresented species. *Aquatic Conservation: Marine and Freshwater Ecosystems* .2020;30(11) : 10.1002/aqc.3410 2050-2065 10.1002/aqc.3410

[2] : Conserving shellfish reefs—a systematic review reveals the need to broaden research efforts. *Restoration Ecology* .2021;29(4) : 10.1111/rec.13375 10.1111/rec.13375

# Response

**Published:** 2022-01-07

**Authors:** Camilla bertolini

**Affiliations:** Ca' Foscari University of Venice, Italy

Reviewer comments in italics throughout.

*In their manuscript “Identifying knowledge gaps for successful restorative aquaculture of Ostrea edulis: a bibliometric analysis” Camila Bertolini and Roberto Pastres seek to identify research gaps in the existing global and local literature surrounding Ostrea edulis, ultimately informing restorative aquaculture projects like MAREA. The concept of the review is compelling and the methods are generally sound, however, the review would greatly benefit from additional information throughout the manuscript. In particular, the introduction would benefit by providing additional background on the study species, MAREA, existing literature reviews, and the specific goals of the project. The results also need clarification around the clustering and subcategorization process. Additionally, the methodology around clustering and keyword analysis needs more information to demonstrate that it is an appropriate study design for this project. Finally, the current conclusions of the review are quite vague. Explicit and detailed recommendations would be very useful to future efforts in this field. Overall, the review has good potential, but the current conclusions and methodology are not supported well enough for the work to have sufficient rigour and ultimately impact for readers and end users.*

**Author response: **We are thankful to the reviewer(s) for the encouraging comments and for their time taken on this manuscript. We have added missing information and details as suggested in all subsections, taking on board all of the specific suggestions (as detailed below each).

***Title and keywords:***

*The title is informative and describes the focus of the review well. Consider replacing the Ostrea edulis keyword as it is already present in the title and will be picked up by search engines.*

**Author response: **Thank you for the title comment. The keywords will be changed during the upload of the corrected manuscript if it will be possible.

***Abstract:***

*The connection between the results and the conclusion is unclear in this abstract. I recommend clarifying the conclusions and more clearly establish the connection to the results.*

**Author response: **This was clarified by changing the conclusions to:

 ‘The review highlighted that restoration success and aquaculture feasibility depend upon the crucial stage of settlement. The project ‘MAREA’ will therefore increase its focus on this stage, both in terms of timing, location and materials for settlement plates placement. ‘

 This better connects it with the results section. 

***Introduction:***

*Further clarification is needed on exactly what “research gaps required for restorative aquaculture” this literature review is targeting. The previous sentence suggests a focus on either ecological gaps (how and where restorative aquaculture can be conducted) or historical baseline gaps (extent of historical populations), but the sentence prior to that suggests a focus on gaps around output quantification (“the relative and absolute success of different strategies”). Is the intention to search for information on all of these gaps or only a narrower scope?*

**Author response: **The intention was to find research that was comprehensive of all of the fields of research, firstly applying a quantitative approach then following with a more qualitative approach, in order to understand where knowledge gaps lie and how do they influence the potential for restorative aquaculture, in order to target them during the project. Details and clarifications were added throughout the introduction with regards to the aim of this work.

*Brief background information on Ostrea edulis would be very helpful in either in the introduction or the beginning of the methods section. This oyster has some unusual aspects to its biology which are particularly pertinent to restoration initiatives. Likewise, some detail of the nature of the biogenic habitat formed by this species would be valuable, and the geographic range of the species.*

**Author response: **We added a paragraph briefly introducing the species:

  ‘There are over 200 species of oysters worldwide, but only 6 species belonging to four main genera ( *Crassostrea, Magallana, Ostrea and Saccostrea*) are currently being commercialised. Species of the genus *Ostrea* have the peculiarity of being ‘brooders’ or ‘partial spawners’ meaning that only males spawn and females brood eggs in their pallial cavity, whereas the other genus are complete spawners where both sexes spawn. This has some implications and reproduction success is dependent upon a threshold density (Guy *et al.*, 2019). *Ostrea edulis*, the European flat oyster, was once widespread in the North Sea, on the Atlantic coast and in other European coastal waters, including the Mediterranean and Black Seas. However flat oysters became functionally extinct in wide areas, mainly due to overfishing, habitat loss, pollution and disease: as a result,  reefs are now one of the most threatened marine habitats in Europe.’  

*Additional information on the methods used here (particularly keyword tracking) would be useful in the introduction. Why is this method being used and what information can be garnered from it?*

**Author response: **We slightly rephrased the methodology section for this part and included a key reference to the package used The package bibliometrix was used for initial automated analyses. The procedures of ‘data collection’ highlighted in (Aria & Cuccurullo, 2017) were followed. For data retrieval both Web of Science and Scopus were searched (all databases, last search 9/07/2021) using the keyword ‘ *Ostrea edulis*’. Titles and abstracts were then independently screened by the author (C.B.) to ensure research was about this species. Only peer reviewed, English language articles were selected in this search. Any duplicate fields were removed.

*Additional background on existing literature reviews of Ostrea edulis research would provide useful information on gaps already known to exist in the literature. The benefits of bivalve reef restoration: A global synthesis of underrepresented species (zu Ermgassen et al., 2020 ^1^ ) documents the ecosystem services provided by Ostrea edulis after a systematic review of the literature while Conserving shellfish reefs – A systematic review reveals the need to broaden research efforts (Toone et al., 2020 ^2^ ) reports on common restoration interventions undertaken for shellfish reefs including Ostrea edulis.*

**Author response:  **Added details and reference to Toone *et al.* in the last paragraph of the intro :

 ‘Most research on restoration, so far, was done in northern America and northern Europe, with Crassostrea virginica dominating and O. edulis taking up  only 1% of the overall research effort (Toone et al., 2021). The objective of this review is the identification of the knowledge gaps to be filled for achieving the goal(s) of MAREA. To this aim, the peer reviewed literature concerning O. edulis was screened using a two step approach: 1) clusters of potentially relevant topics were identified using automated bibliographic tool which , based on a set of keywords (Aria & Cuccurullo, 2017) and then 2) going into details of each cluster identified previously using both automated tools and manual searches. This helped to narrow the focus of the data gathering of the project MAREA, making optimal use of available knowledge to maximize the use of the funds available. Moreover, since the study by Toone *et al.* (2021) highlighted how only 2% of the knowledge is from indigenous sources, likely leading to an evidence base that is not reflective of the circumstances for most potential shellfish conservation projects, .ultimately imperilling the success of their conservation work. A local knowledge section including both historical and modern sources was therefore added with searches in Italian.’

*MAREA is mentioned in the abstract and conclusion, but never actually introduced anywhere in the manuscript, which seems a lost opportunity. Consider adding information on this project to the introduction.*

**Author response: **We added details in the introduction:

**‘MAREA aims at assessing the feasibility of restoring flat oyster reefs in the Northern Adriatic, by taking into account  the ecological constraints and potential for improving the overall ecosystem functioning , as well as the  economic benefits for shellfish farmers. The economic output and involvement of the aquaculture sector as a stakeholder in restoration should help securing financial resources to allow for large scale restorative actions (Fitzsimons *et al*., 2020) […]The objective of this review is the identification of the knowledge gaps to be filled for achieving the goal(s) of MAREA […]This helped to narrow the focus of the data gathering of the project MAREA, making optimal use of available knowledge to maximize the use of the funds available.’

***Methods:***

*Clarification is needed in the fifth paragraph on whether ‘restoration’ and ‘aquaculture’ were ran through Web of Science and Scopus as new search terms or whether the already identified papers were then further narrowed down by these search terms.*

**Author response: **Clarification on how the “broad topics” articles used for article sorting were developed would be useful. Were they pre-defined or developed after reading? The methods section now includes this clarification, specifying that was done after reading

*Why is the keyword clustering method being used? What information does it present that helps address the goals of this review? Added some of this in the introduction.*

**Author response: **The objective of this review is the identification of the knowledge gaps to be filled for achieving the goal(s) of MAREA. To this aim, the peer reviewed literature concerning O. edulis was screened using a two-step approach: 1) clusters of potentially relevant topics were identified using automated bibliographic tool which , based on a set of keywords (Aria & Cuccurullo, 2017) and then 2) going into details of each cluster identified previously using both automated tools and manual searches.**

*Were repeats present between the global knowledge and local knowledge searches?*

**Author response: **The local knowledge database search appears to just be a more narrow repeat of the same search conducted earlier (any papers with the keywords ‘ *Ostrea edulis*’ and one of the location specific terms should have already shown up under just ‘ *Ostrea edulis*’).

*Was the ‘local knowledge’ database search also conducted in Italian to initiate new results or only the library search?*

**Author response: **This search differed as  different sources were also included (Google Scholar, library catalogue, reference list from local institution)  and in particular the search was repeated in Italian, therefore more literature was found (which was primarily in Italian).  

*How were the papers that resulted from the ‘local knowledge’ search analysed? Identically to the global knowledge results or using different methods? This information should be added to the ‘Local knowledge’ subsection.*

**Author response: **As we now have specified the local knowledge yielded results that were slightly different in typology from the global analysis, a few journal articles but mostly reports and chapters in books. Therefore, the analysis was primarily of a qualitative nature after thoroughly reading the material to extract information related to ecological status and aquaculture/restoration efforts.

***Results and discussion:***

*As a whole this section is very light on discussion and could benefit from a more in-depth insight into some of the implications of these findings. Separate results and discussion sections may prove useful.*

**Author response: **We added details throughout but kept the one section, in order to avoid repetition and keep the story fluent.

*The geographic results of this review are interesting, but difficult to ascertain in their current form. What are the numbers behind “Geographically, studies were primarily from Atlantic and North Sea regions” or “studies spanned both Atlantic and Mediterranean European regions”? A map of included studies as a figure may be a very useful addition in this regard.*

**Author response: **A map (figure 3) was added following this suggestion

*The connection between Figure 2 and Table 1 is unclear. How do the subclusters identified in Table 1 correlate with the clades in Figure 2? For example, there’s only two clear clades in cluster three (red) but three subclusters. How do the terms used to identify the leaves in the dendogram correlate with the words characterizing each subcluster? More information on these connections would be very informative.*

**Author response: **Thank you for pointing out that the connection between the dendogram and the table was not as clear for the reader. We now specify better in the text that while the ‘clusters’ in the table are the 3 clusters in the dendogram, the ‘sub-clusters’ are based on the title and abstract clustering analysis of the papers within each sub-group (see updated legend of table 1 which now reads *‘Subcluster characterization. Words in title, keywords and abstract characterizing each sub cluster’ *). This was done to avoid relying only on the keywords alone, which can be useful to have a first distinguish (otherwise too many ‘words’ are found which can give meaningless outputs) but the inclusions of titles  and abstracts can help in more in depth topic analyses.

*Discussion on how the identified clustering informs restorative aquaculture decisions is needed. Where are the current gaps in the research? What does the clustering tell us about how to move forward with restorative aquaculture projects?*

**Author response: **The results and discussion section was integrated throughout, and a part was added to say:

 ‘This clustering analysis revealed some interesting patterns highlighting some of the main issues that may be considered by both restoration and aquaculture of this species. Infection, particular bonamiosis, may be an obstacle to both activities…’

*Why was the clustering method only used for 156 out of the 514 papers? Did the remaining papers have no common terms? Clarification would be useful.*

**Author response: **The min degree setting of the Conceptualstructure was already explained in the methods. This was clarified in the discussion:

 ‘The clustering method used only 156 (30%) papers with common keyword associations, meaning the other papers did not share important terms (appearing at least 10 times) in their keywords.’

*There are 32 identified restoration articles, but the more detailed breakdown in the seventh paragraph reports 35 articles. Is this because a single article could be categorized into multiple themes? Did only three articles fit into multiple categories? Clarification in the methods section would be helpful.*

**Author response: **32 was a typo which should in fact read 35 and it has now been changed.

*The information presented in the seventh paragraph identifying ecological parameters for successful populations is very useful. Consider making this into a table to emphasize these findings and allow for easier interpretation.*

**Author response: **This paragraph was now modified changed into a table 2 introduced as ‘The restoration papers dealing with ecological understanding, for example, presented some agreements with regards to the value of the main environmental parameters necessary for success, as summed up in table 2.’

*Was it possible for the aquaculture papers to be subcategorized into multiple groups in the eighth paragraph and none were or was each paper intentionally only assigned a single subcategory?*

**Author response: **We made this clearer by adding ‘ *being assigned to a single sub-category after reading’*.

*The local knowledge section is very informative. Consider making additional comparisons between this historical local knowledge and the results of the current review as there seem to be interesting similarities and differences.*

**Author response: **Thank you for this remark. Instead of adding the comparisons in the local knowledge section we took the suggestion in this comment and expanded the conclusion section. E.g. at the end of the first paragraph we added:

 “What emerges in particular is the concern for the substrate limitation which was already clear from the local historical perspectives and arises also as one key issue in the modern global restoration literature (Bromley *et al*., 2016; Chapman *et al*., 2021; Rodriguez-Perez *et al*., 2019; Smyth *et al*., 2018). Further, the account of optimal substrates in the historical literature seems, at least in part (not too muddy), in accordance with current literature (table 2).”

***Conclusions:***

*This conclusion section is very brief and would benefit greatly from additional details.*

**Author response: **At the end of the first paragraph we added some details to the importance of consulting “grey” historical literature (like the case of most “local” knowledge found in the current study):

 ‘This implies that the amount of knowledge present in what may be considered as “grey literature” may be greater than imagined and already contain some essential information that could move restoration forward at a faster rate and with a greater chance of success. Of course, to access this it is important to access all repositories in multiple language and may require therefore international collaborations.’

*The conclusion states “…the collated information on the ecological drivers, such as those presented in the review regarding Ostrea edulis should be used…” but does not address how this information should be used.*

**Author response: **This paragraph implied that it should be used in the drawing of restoration plans. This is now made more explicit.

*These results “led to the identification of a suitable area within the Venice lagoon to conduct the pilot” for MAREA, but the conclusion does not state which results led to this ability or how they were used. Were suitable areas compared with the ecological information? Was the local knowledge used to identify historical areas that could be used? How could another project use this information in a similar way?*

**Author response: **We added some information related to this aspect :

 ‘In this study we noticed how local historical knowledge presented some similarities with recent, more globalised, studies, pointing to the potential ubiquitousness of some of the issues related to substratum availability and how these, despite being known since the late 1800s, have not yet been resolved and may still be the main issue in the Venice Lagoon, the area where MAREA is taking place. However, local studies in other areas may present other issues as priority (e.g. temperature or current) and therefore it would be recommended to delve deeper into the historical reasons for disappearance and lack of natural re-establishment before embarking in restoration.’

*Finally, the conclusion recommends that “this knowledge should be convened in appropriate manners to aquaculture practitioners, bridging linguistic gaps, in order to make the two worlds coexist and limit the possibility of failed attempts.” How would the authors suggest this is done? What are the “appropriate manners” and what exactly is the knowledge the authors are suggesting is shared?*

**Author response: **We rephrased this part to make it clearer:

 ‘Furthermore, the knowledge relating to seed production and harvesting learnt from academic and restoration trials, should be convened in appropriate manners, this means bridging linguistic gaps and jargon,  to aquaculture practitioners, bridging linguistic gaps, in order to make the two worlds, research and practice, coexist and limit the possibility of failed attempts.’

*This review provides a lot of interesting results, but these recommendations need to be streamlined to clarify how the authors think this information will be more useful.*

**Author response: **Thanks for this kind comment and for appreciating the study,

***Figures and tables:***

*Consider adjusting the y-axes of the two inset graphs in Figure 1 to be identical (0-15). In the figure’s current form it appears the aquaculture and restoration subtopics contain similar numbers of articles, when aquaculture actually includes fewer articles than restoration but has a smaller scale on the y-axis.*

**Author response: **Thank you for this. Figure 1 (now figure 2) was now changed and both y axis of the two in-sets are on the same scale. Now the x axis is also the same scale for all three graphs.

*Does the “Height” y-axis of Figure 2 correlate to any real values? I would suggest simply removing this axis.*

**Author response: **This axis was removed as suggested

***Typos:***

*Abstract under the results subsection: “While diseases was a leading cause for reef loss…” should be “While diseases were a leading cause for reef loss”*

**Author response: **Thank you for noticing this, it was changed

*First sentence of introduction: “…which are important habitats supporting the biodiversity of marine ecosystems and contribute to multiple ecosystem services…” should either be “…which are important habitats that support the biodiversity of marine ecosystems and contribute to multiple ecosystem services…” or “…which are important habitats supporting the biodiversity of marine ecosystems and contributing to multiple ecosystem services…”*

**Author response: **Thank you for this English suggestion, it was now changed to the first

*First sentence of the third paragraph of the introduction: “Bivalve aquaculture has in recent years returned in the first line with an emphasis on sustainability.” What is the meaning of “returned in the first line”?*

**Author response: **This was changed to read ’Bivalve aquaculture has in recent years appeared as a solution for protein production with an emphasis on its sustainability’.

*The placement of the “(Figure 1)” citation in the first paragraph of the results section is unusual and breaks up the sentence. Consider moving it to the end of the sentence.*

**Author response: **This is now being placed at the end of the sentence.

***References:***

*Burrell (1983) is entirely capitalized.*

*Check capitalization consistency throughout references as some use title case and others use sentence case.*

*Jones (1994) has extra word “organisms”.*

**Author response: **Thank you for noticing these typos and these two references have now been corrected.
